# Vitamins as Modulators of Neurodegenerative Disease Pathways: Mechanisms and Therapeutic Perspectives

**DOI:** 10.3390/nu18060995

**Published:** 2026-03-20

**Authors:** Karolina Kwaśniewska, Weronika Fic, Ewelina Polak-Szczybyło

**Affiliations:** 1 Student Scientific Club of Human Nutrition, Faculty of Health Sciences and Psychology, Collegium Medicum, University of Rzeszów, ul. Warzywna 1a, 35-959 Rzeszow, Poland; kk130958@stud.ur.edu.pl (K.K.); wf130811@stud.ur.edu.pl (W.F.); 2 Faculty of Health Sciences and Psychology, Collegium Medicum, University of Rzeszów, ul. Warzywna 1a, 35-959 Rzeszow, Poland

**Keywords:** neurodegenerative diseases, vitamins, oxidative stress, neuroinflammation, Alzheimer’s disease, Parkinson’s disease

## Abstract

Neurodegenerative diseases, including Alzheimer’s disease, Parkinson’s disease, and amyotrophic lateral sclerosis, currently represent one of the major challenges in contemporary medicine and geriatrics. Progressive degeneration of the nervous system affects not only patients’ physical functioning but also their psychosocial well-being, often leading to social isolation and disruption of interpersonal relationships. These processes are most strongly associated with individuals over 65 years of age, in whom metabolic syndrome is frequently diagnosed and constitutes a significant factor predisposing them to the exacerbation of neuropathological changes. This review analyzes the role of selected vitamins in modulating the course of neurodegenerative disorders, with particular emphasis on their neuroprotective potential. Specific attention is given to their involvement in antioxidant defense mechanisms, regulation of inflammatory pathways, prevention of abnormal protein aggregation, participation in neurotransmitter synthesis, and support of mitochondrial function and cellular energy metabolism. The review also considers key interactions between vitamins and coenzyme Q10, which synergistically enhance neuroprotective mechanisms. Deficiencies in certain vitamins may exacerbate oxidative stress, impair synaptic transmission, and intensify neuroinflammatory responses, thereby contributing to disease progression. The study analyzes the available data on therapeutic doses of vitamins and compares them with the recommended dietary intake and the upper tolerable intake levels (UL). The available evidence suggests that personalized vitamin supplementation, when integrated with a well-balanced and nutrient-dense diet, may constitute a valuable adjunctive therapeutic strategy. Such an approach may help attenuate disease progression, support neuronal integrity, and improve functional outcomes. Ultimately, targeted nutritional interventions may enhance overall well-being and quality of life in patients affected by neurodegenerative diseases.

## 1. Introduction

Neurodegenerative diseases constitute a heterogeneous group of nervous system disorders characterized by the selective and progressive loss of neuronal populations, ultimately leading to the irreversible dysfunction of neural structures. According to the World Health Organization (WHO) data from 2021, neurological disorders affect more than 3 billion people worldwide and represent one of the leading causes of long-term disability. The pathogenesis of these conditions—encompassing oxidative stress, neuroinflammation, and apoptosis—is closely associated with the aging process [1,2,3,4]. Although a higher overall incidence is observed in men, certain phenotypes, such as dementia, demonstrate greater prevalence among women [3]. A critical factor in the development of neurodegenerative disorders, including Alzheimer’s disease (AD), Parkinson’s disease (PD), and amyotrophic lateral sclerosis (ALS), is age-related mitochondrial dysfunction. This dysfunction disrupts bioenergetic homeostasis, promotes excessive production of reactive oxygen species, and impairs proteostasis mechanisms, resulting in pathological protein aggregation and nucleic acid damage [5,6]. The presence of metabolic syndrome frequently exacerbates these processes, acting as a mediator that intensifies neuropathological alterations. Given the growing public health burden posed by neurodegenerative diseases, increasing attention is being directed toward strategies aimed at modulating their progression through nutritional interventions. Emerging evidence suggests that supplementation with selected vitamins exhibiting neuroprotective properties may effectively support mitochondrial function and neurotransmitter biosynthesis. The implementation of targeted micronutrient intake as part of adjunctive therapy, combined with a balanced and nutrient-dense diet, shows promise in slowing disease progression and improving patients’ overall quality of life.

### 1.1. Alzheimer’s Disease

Alzheimer’s disease (AD) is the leading cause of dementia in the geriatric population. According to the Alzheimer’s Association, approximately 7.2 million individuals aged 65 years and older in the United States are projected to be living with AD in 2025, with a marked predominance among women, who account for nearly two-thirds of cases [7]. Beyond advanced age, a key genetic determinant of AD is the presence of the apolipoprotein E ε4 allele (APOE ε4), which is strongly associated with increased deposition of beta-amyloid aggregates and the formation of characteristic senile plaques within brain tissue [8]. Current clinical diagnosis is based on the 2011 National Institute on Aging–Alzheimer’s Association (NIA-AA) guidelines, which categorize the level of diagnostic certainty as definite (histopathological confirmed), probable, possible, or unlikely AD. These criteria incorporate advances in biomarker analysis as well as comprehensive neuropsychological assessment [9]. The etiology of AD is multifactorial and involves complex interactions among aging processes, genetic predisposition, environmental influences, and infectious factors [9,10,11]. In its advanced stages, the disease leads to profound deterioration of cognitive and motor functions, ultimately resulting in complete loss of independence, inability to recognize close relatives, progressive dysphagia, and severe functional decline. The cumulative progression of these pathological processes ultimately contributes to the fatal course of the disorder.

### 1.2. Parkinson’s Disease

PD is the second most prevalent neurodegenerative disorder worldwide and is characterized by the fastest growing incidence rate among such conditions. Epidemiological data from the United States indicate approximately 90,000 newly diagnosed cases annually [12]. The occurrence of PD increases with age, reaching its peak in individuals over 65 years. Prevalence rises progressively with advancing age, with a marked predominance among men, which has been partly attributed to the absence of the neuroprotective effects of estrogen in this population [13]. The etiology of PD remains incompletely understood, although interactions between genetic susceptibility and environmental exposures are considered central to disease development [14]. The key pathophysiological mechanism involves the progressive and irreversible degeneration of dopaminergic neurons in the substantia nigra pars compacta, as well as in the mesocorticolimbic system and the hypothalamus. Dopamine deficiency gives rise to the classical motor symptom triad: bradykinesia, resting tremor—most commonly affecting the upper limbs—muscle rigidity, and postural instability. However, the clinical spectrum of PD extends beyond motor dysfunction. Non-motor manifestations are common and include olfactory impairment, autonomic dysfunction, cognitive decline, chronic pain syndromes, and persistent fatigue. As the disease progresses, these symptoms intensify, leading to significant functional impairment and a gradual loss of patient autonomy [15].

### 1.3. Amyotrophic Lateral Sclerosis

ALS is a rare, progressive, and ultimately fatal neurodegenerative disorder characterized by the degeneration of motor neurons within the central nervous system. The disease most commonly affects individuals over the age of 55 and demonstrates approximately twice the incidence in men compared to women. The global incidence is estimated at 1–3 cases per 100,000 population annually [16,17]. The etiology of ALS remains incompletely understood. While a proportion of cases are linked to identifiable genetic mutations, the majority are sporadic, with current hypotheses emphasizing the interaction of genetic susceptibility and environmental or toxic exposures as potential contributing factors [18]. Early clinical manifestations typically include muscle fasciculations, paresthesia, and progressive weakness of the distal limbs, as well as dysphagia and dysarthria. As the disease advances, widespread muscle atrophy develops, leading to profound motor impairment and progressive loss of voluntary muscle control. Autonomic dysfunction may also occur, particularly involving swallowing and respiratory function. Ultimately, respiratory failure represents the leading cause of mortality in affected individuals. The diagnosis of ALS requires a multidisciplinary approach, incorporating detailed neurological examination, electrophysiological studies, laboratory testing, and magnetic resonance imaging to exclude other neuromuscular or neurodegenerative conditions [16,18].

## 2. Methods

This study is a narrative review aimed at providing a synthetic overview of the current state of knowledge regarding the role of vitamins as modulators of neurodegenerative pathways, with particular emphasis on their mechanisms of action, their importance for nervous system function, and their potential therapeutic applications in neurodegenerative diseases. Additionally, the review describes key interactions between vitamins and coenzyme Q10 in the nervous system and identifies existing research gaps as well as potential future research directions.

The literature search was conducted between November 2025 and March 2026 using the electronic databases PubMed and Google Scholar. The search strategy included combinations of English-language keywords used individually and in combination with logical operators (“AND”, “OR”).

The following terms were among those used in the search process: neurodegeneration, neurodegenerative diseases, Alzheimer’s disease, Parkinson’s disease, amyotrophic lateral sclerosis, vitamins and neurodegenerative diseases, vitamin D and neurodegenerative diseases, B vitamins and neurodegenerative diseases, vitamin E and neurodegenerative diseases, vitamin A and neurodegenerative diseases, vitamin C and neurodegenerative diseases, vitamin K and neurodegenerative diseases, coenzyme Q10 and neurodegenerative diseases, vitamin interactions, neuroprotective mechanisms of vitamins and coenzyme Q10, and preventive and therapeutic strategies in neurodegenerative diseases.

Publications published in English between 2015 and 2026 were included in the analysis. In justified cases, earlier studies were also considered when they were essential for understanding the pathophysiology of neurodegenerative diseases or the mechanisms of action of vitamins.

The review included studies describing the pathophysiology of neurodegenerative diseases, the effects of vitamins on the nervous system, neurotransmitter synthesis, mitochondrial function, and neuroinflammatory processes, as well as studies examining vitamin interactions in the context of neuroprotection. Clinical studies, observational studies, and experimental studies conducted on animal models evaluating the influence of vitamins on the development of neurodegenerative diseases were also included.

The study selection process involved the manual screening of titles, abstracts, and full-text articles by the authors. Editorials, letters to the editor, and publications without access to the full text were excluded from the review.

## 3. Pathophysiology of Neurodegenerative Diseases

### 3.1. Pathophysiology of Alzheimer’s Disease

**Figure 1:**
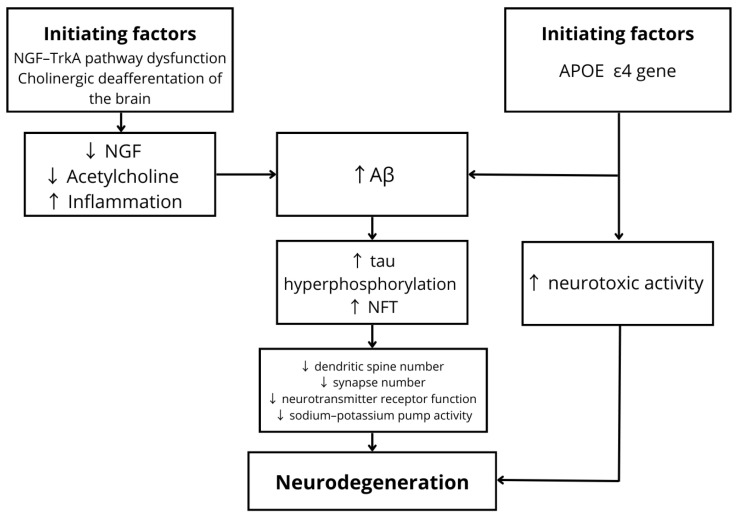
Pathogenesis of Alzheimer’s disease. Disruption of the NGF–TrkA signaling pathway leads to decreased acetylcholine levels, increased neuroinflammation, and enhanced β-amyloid (Aβ) accumulation. The APOE ε4 allele promotes Aβ deposition and exerts independent neurotoxic effects. Disease progression is associated with increased tau hyperphosphorylation and formation of neurofibrillary tangles (NFTs). These processes contribute to reduced dendritic spine density, decreased synaptic number, and impaired function of neurotransmitter receptors and the Na^+^/K^+^-ATPase, ultimately leading to progressive neurodegeneration. Abbreviations: Aβ—beta-amyloid; NFT—neurofibrillary tangle; ↑ increase; ↓ decrease.

AD is an irreversible disorder characterized by progressive neurodegenerative processes [19], clinically manifested by dementia, cognitive dysfunction, and apathy [19,20,21,22,23,24,25,26]. In the early, preclinical stages of the disease, progressive damage to cholinergic neurons occurs, leading to cholinergic deafferentation of the hippocampus, cerebral cortex, and amygdala [20]. Disruption of the Nerve Growth Factor–Tropomyosin receptor kinase A (NGF–TrkA) signaling pathway reduces the availability of NGF to cholinergic neurons, resulting in decreased acetylcholine levels and accelerated neurodegeneration. This process is accompanied by enhanced neuroinflammation and increased accumulation of beta-amyloid peptide (Aβ) [19]. A key genetic factor in AD pathogenesis is the apolipoprotein E (APOE) gene, particularly the ε4 allele (APOE ε4), which significantly increases the risk of disease development. Under physiological conditions, APOE is involved in lipid transport within the brain. However, in pathological states, it promotes the formation and deposition of Aβ aggregates, a process further intensified by the presence of APOE ε4 [24]. Moreover, APOE ε4 exerts neurotoxic effects independent of Aβ pathways by reducing the number of γ-aminobutyric acid (GABA)-ergic interneurons in the hippocampus, thereby impairing neuronal function [26]. Beta-amyloid peptides generated through sequential cleavage of amyloid precursor protein (APP) by the beta-site APP cleaving enzyme 1 (BACE-1) complex [21] contribute to neurodystrophic damage and synaptic loss. Aβ monomers disrupt neuronal connectivity by impairing neurotransmitter receptor function and inhibiting sodium–potassium pump activity [22]. Early stages of dementia are further exacerbated by dysregulation of the balance between Low-density lipoprotein receptor-related protein 1 (LRP-1) and Receptor for Advanced Glycation End Products (RAGE) [19]. As pathological processes progress, hyperphosphorylation of tau protein leads to the formation of intracellular neurofibrillary tangles (NFTs). Accumulation of NFTs within the entorhinal cortex and hippocampus [22] results in dendritic spine loss, synaptic reduction [23], neuronal death, and progressive brain atrophy [22]. Despite significant advances in research, the pathophysiology of AD remains incompletely understood, underscoring the need for further investigation [23]. The pathogenesis of Alzheimer’s disease is shown in Figure 1.

### 3.2. Pathophysiology of Parkinson’s Disease

**Figure 2:**
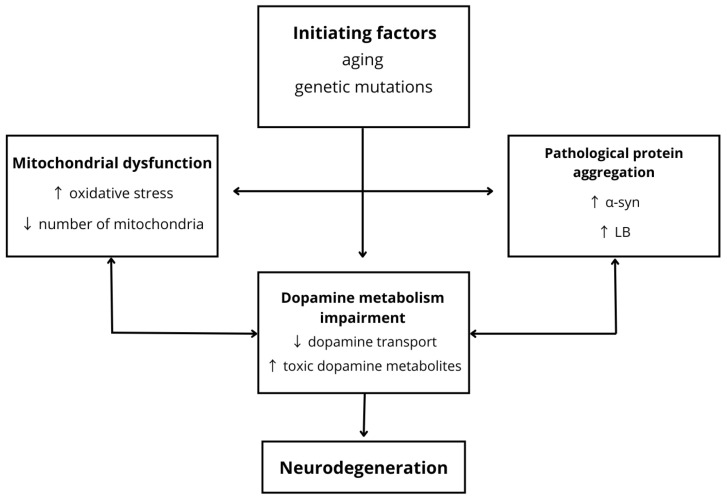
Pathogenesis of Alzheimer’s disease. Disruption of the NGF–TrkA signaling pathway leads to a decrease in acetylcholine levels, an increase in neuroinflammation, and increased accumulation of beta-amyloid (Aβ). The APOE ε4 allele enhances Aβ deposition and exerts independent neurotoxic effects. As the disease progresses, increased tau hyperphosphorylation and formation of neurofibrillary tangles (NFTs) occur. These processes result in a reduction in dendritic spine density, decreased synaptic number, and impaired function of neurotransmitter receptors and the sodium–potassium pump, ultimately leading to progressive neurodegeneration; ↑ increase; ↓ decrease.

PD is a neurodegenerative movement disorder [27] characterized by rigidity, resting tremor, postural instability, and bradykinesia [28]. In the preclinical phase, disturbances in presynaptic dopaminergic activity occur as a result of genetic alterations. Mutations such as Vps35-D620N reduce dopamine release, as demonstrated by microdialysis studies, while the G2019S mutation inhibits dopamine secretion. Alterations in the Parkin gene contribute to mitochondrial dysfunction, leading to impaired dopamine release. Furthermore, overexpression of the SNCA-A53T mutation in PITX3 dopaminergic neurons initiates neuronal damage and motor dysfunction [29]. Impaired transport of dopamine into synaptic vesicles [30], which become damaged under the influence of alpha-synuclein (α-Syn) aggregates [31], results in cytoplasmic accumulation of the neurotransmitter and the formation of neurotoxic metabolites with neurodegenerative properties [29]. Among these, DOPAL (3,4-dihydroxyphenylacetaldehyde) [27] is particularly significant. Its accumulation in the substantia nigra pars compacta (SNpc) disrupts neuronal proteostasis. Moreover, this metabolite promotes increased oxidative stress within mitochondria and activates mechanisms of necrosis and apoptosis [32]. The development of neurodegenerative pathology gradually spreads across different regions of the brain. It is suggested that disease progression may result from the differential vulnerability of central and peripheral structures to the pathological process, as well as from the distinct compensatory capacities of specific neuronal populations. However, Parkinson’s disease (PD) primarily affects the substantia nigra pars compacta (SNpc) [33] and the putamen [34], regions rich in dopaminergic neurons, leading to characteristic motor symptoms such as tremor, rigidity, and postural instability. The mechanisms responsible for the exceptional vulnerability of SNpc dopaminergic neurons remain unclear. It is hypothesized that these neurons possess an intrinsically limited calcium-buffering capacity and a predisposition to pacemaking activity. Moreover, dopaminergic neurons are responsible for the metabolism and degradation of dopamine, a neurotransmitter synthesized and released within the nigrostriatal pathway, which makes them particularly susceptible to the toxic dopamine metabolite DOPAL [33]. Dysfunction of the ubiquitin–proteasome system [31], together with enhanced α-syn oligomerization induced by DOPAL [29], facilitates the accumulation of pathological protein aggregates, ultimately leading to the formation of Lewy bodies (LB). Lewy bodies predominantly localize within axonal pathways and synaptic terminals, contributing to mitochondrial dysfunction, reduced synthesis of cholinergic and cate-cholaminergic neurotransmitters [34], gliosis, neuronal loss [28], and the development of dementia [34]. The lack of sufficient studies describing the mechanisms of action of DOPAL in other specific brain regions represents a significant obstacle to fully understanding its role in neurodegenerative processes. Moreover, another major challenge for the scientific community is to explain why mutations in different proteins, with diverse or not yet fully understood functions, lead to similar pathological phenotypes, also observed in idiopathic Parkinson’s disease. Further research is therefore required to clarify these mechanisms [33]. Figure 2 presents a schematic overview of the pathophysiology of PD.

### 3.3. Pathophysiology of Amyotrophic Lateral Sclerosis

**Figure 3:**
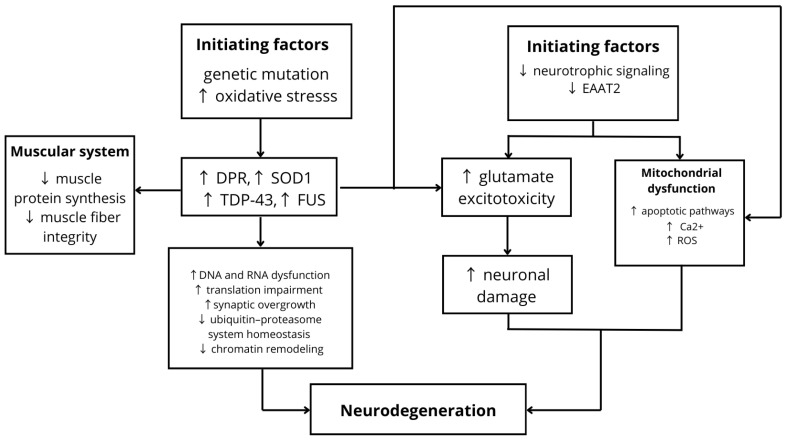
Genetic mutations and oxidative stress lead to the aggregation of DPR, SOD1, TDP-43, and FUS, resulting in DNA and RNA dysfunction, impaired translation, synaptic alterations, disruption of ubiquitin–proteasome system homeostasis, and chromatin remodeling. At the same time, abnormally aggregated proteins exacerbate glutamate excitotoxicity and mitochondrial dysfunction, including activation of apoptotic pathways, increased production of reactive oxygen species (ROS), and impaired Ca^2+^ buffering, associated with reduced neurotrophic signaling and decreased levels of EAAT2. In addition, aberrant protein aggregation contributes to reduced synthesis of muscle proteins and muscle fiber atrophy; ↑ increase; ↓ decrease.

ALS is a fatal neurodegenerative disease characterized by motor, neurobehavioral, and cognitive dysfunction [35]. In the early stages of the disorder, impairments in axonal transport, loss of neurotrophic signaling, and reduced expression of Excitatory Amino Acid Transporter 2 (EAAT2) are observed. This leads to the accumulation of glutamate in the extracellular space, resulting in excitotoxic neuronal damage, excessive activation of glutamate receptors and Ca^2+^-dependent enzymatic pathways, and increased production of reactive oxygen species (ROS) [36]. Simultaneously, disruption of intracellular Ca^2+^ buffering mechanisms promotes mitochondrial membrane depolarization and activation of apoptotic pathways [37]. Additionally, decreased transcription of genes encoding potassium channels contributes to neuronal hyperexcitability [38]. It should also be emphasized that ALS may have a genetic basis. An expanded hexanucleotide repeat (GGGGCC) in the first intron of the C9ORF72 gene undergoes repeat-associated non-AUG (RAN) translation, resulting in the production of neurotoxic dipeptide repeat proteins (DPRs). The accumulation of these proteins contributes to DNA damage, impaired RNA processing and translation, and disruption of nucleocytoplasmic transport. Consequently, glutamate excitotoxicity is further exacerbated, synaptic hypertrophy occurs, the number of active zones is reduced, and progressive motor neuron degeneration ensues [35]. Mutations in the superoxide dismutase 1 (SOD1) gene lead to protein misfolding and its pathological accumulation in cells [37]. Aggregation of SOD1 disrupts Ca^2+^ and redox homeostasis, which contributes to mitochondrial dysfunction. Interestingly, aggregated forms of mutant SOD1 can propagate between cells and spread across different regions of the central nervous system (CNS) in a prion-like manner, leading to damage of vulnerable neuronal populations [39]. Mutations in exon 6 of the TARDBP gene promote the loss of functional TDP-43 and its increased aggregation in cytoplasmic inclusion bodies in the brain and spinal cord neurons. TDP-43 may form spherical and ring-like oligomers, which exhibit cytotoxic effects on neurons. Additionally, TDP-43 aggregation may be promoted by impaired ubiquitin–proteasome system homeostasis. Abnormal accumulation of this protein results in RNA metabolism dysregulation, mitochondrial dysfunction, inhibition of protein degradation, disturbances in endocytosis, and chromatin remodeling [40]. Similarly, mutations in the FUS gene result in aggregation of the FUS protein, leading to dysregulation of RNA processing and enhanced neurodegeneration [38]. Moreover, oxidative stress promotes the aberrant aggregation of SOD1, TDP-43, and FUS [39]. Alterations in SOD1, TDP-43, and FUS lead to reduced synthesis of muscle proteins [41,42], while dipeptide repeat proteins (DPRs) contribute to muscle fiber atrophy in amyotrophic lateral sclerosis (ALS) [42]. Figure 3 presents a schematic illustration of the pathophysiology of ALS.

## 4. Impact of Vitamins on the Nervous System, Neurotransmitter Synthesis, and Mitochondrial Function

Vitamins play roles far more complex than merely supporting basic biological functions in the human body. They are particularly essential for the health and proper functioning of the nervous system, where their activity is based on several fundamental mechanisms, including the facilitation of mitochondrial energy production, support of neurotransmitter synthesis, and protection against oxidative stress [43].

### 4.1. B-Complex Vitamins

The B-complex vitamins comprise eight water-soluble micronutrients. The human body is unable to synthesize or store most B vitamins, making continuous dietary intake essential. These vitamins collectively play crucial roles in maintaining nervous system function due to their participation in key biochemical processes within neurons. They are involved in neuronal energy metabolism, neurotransmitter synthesis, myelin sheath protection, gene regulation, and methylation processes. Deficiencies are strongly associated with neurological and neuropsychiatric disorders [44].

#### 4.1.1. Vitamin B_1_ (Thiamine)

Thiamine, an exogenous nutrient, must be regularly supplied through a diet rich in whole grains, pork, and selected vegetables (e.g., potatoes, spinach, parsley), as well as fruits [44,45]. Its biological activity depends on its conversion to thiamine pyrophosphate (TPP), which serves as a crucial coenzyme in the energy metabolism of carbohydrates, lipids, and proteins. TPP acts as a catalytic cofactor for key enzymatic complexes, including pyruvate dehydrogenase, α-ketoglutarate dehydrogenase, and transketolase. These enzymes function at the intersection of glycolysis, the Krebs cycle, and the pentose phosphate pathway, enabling efficient biosynthesis of adenosine triphosphate (ATP) and nicotinamide adenine dinucleotide (NADH) [45,46,47]. The role of thiamine extends beyond strictly metabolic functions, encompassing a critical influence on nervous system homeostasis. It participates in myelin synthesis and neurotransmitter production, and regulates nerve impulse conduction through activation of chloride channels and stabilization of cellular membranes. Thiamine deficiency triggers a cascade of biochemical disturbances, including mitochondrial dysfunction and impaired glucose metabolism. Clinically, this condition manifests as cognitive decline, memory impairment, chronic fatigue, sleep disturbances, and mood disorders. Prolonged vitamin B_1_ deficiency is recognized as a significant etiological factor in the pathogenesis of neurodegenerative diseases [48].

#### 4.1.2. Vitamin B_9_ (Folate)

Folic acid and its active derivatives are essential cofactors in processes that ensure the proper development and functioning of the central nervous system (CNS). Their critical role stems from participation in one-carbon metabolism, which is directly linked to the biosynthesis of purine and pyrimidine nucleotides as well as the production of key neurotransmitters. During the prenatal period, adequate folate intake is crucial for neurulation; supplementation with folic acid in pregnant women significantly reduces the risk of neural tube defects in the fetus, confirming its fundamental embryological importance [49]. An important aspect of vitamin B_9_ biological activity is the regulation of metabolic homeostasis through control of plasma homocysteine levels. Folate deficiency leads to hyperhomocysteinemia, which induces oxidative stress and neurotoxic processes, particularly within dopaminergic neurons. This mechanism is closely associated with the etiopathogenesis of Parkinson’s disease. Studies indicate that disturbances in the folate cycle are linked to alterations in DNA methylation profiles, potentially resulting in epigenetic dysregulation of genes involved in neuronal survival and the development of neurodegenerative disorders [50]. Recent findings suggest that the genomic effects of folic acid are highly precise and dose-dependent, as well as influenced by offspring sex. Although moderate supplementation during pregnancy is essential for preventing developmental defects, it may also induce sex-specific changes in gene expression in embryonic and infant brains, highlighting the role of folates as critical modulators of neurodevelopment [51].

#### 4.1.3. Vitamin B_12_ (Cobalamin)

Vitamin B_12_ is a critical micronutrient functioning as a cofactor in fundamental enzymatic reactions that maintain metabolic and genetic homeostasis. Its role is primarily centered on two key processes: the cytoplasmic synthesis of methionine from homocysteine (in cooperation with folate) and the mitochondrial conversion of methylmalonyl-CoA to succinyl-CoA. The latter reaction, dependent on cobalamin, is essential for the proper functioning of the Krebs cycle and efficient cellular energy metabolism [52]. Cobalamin is indispensable for nucleotide synthesis, thereby ensuring the fidelity of DNA replication and transcription. Vitamin B_12_ deficiency induces genomic instability, leading to DNA strand breaks and impaired methylation processes. In addition, cobalamin exhibits significant antioxidant properties, protecting chromatin structure from cytotoxic damage induced by reactive oxygen species (ROS). These protective effects, demonstrated in both in vivo and in vitro models, underscore the multifaceted role of vitamin B_12_ in cellular defense mechanisms [53]. Deficiency of vitamin B_12_ extends beyond simple metabolic disturbances and results in complex neurological decompensation. Molecular and cellular consequences of hypovitaminosis include not only demyelination but also profound alterations in intercellular signaling and neuroinflammation. Clinically, this manifests as a broad spectrum of disorders ranging from peripheral neuropathies and neurodegenerative diseases to neuropsychiatric symptoms and cognitive impairment [54].

### 4.2. Vitamin C (Ascorbic Acid)

Ascorbic acid is a key water-soluble micronutrient with systemic activity that reaches concentrations in brain tissue significantly higher than those observed in other organs. Its accumulation in neurons is an active process mediated by the specific sodium-dependent vitamin C transporter 2 (SVCT2), highlighting the fundamental importance of this molecule for neural tissue metabolism [55]. The neuroprotective properties of vitamin C are primarily attributed to its potent antioxidant activity. It effectively scavenges reactive oxygen species (ROS), thereby protecting DNA from oxidative mutations and preserving mitochondrial integrity against oxidative stress. In addition, ascorbic acid plays a significant role in neurodevelopmental processes by supporting myelin synthesis, promoting neuronal differentiation, and positively modulating immune responses within the central nervous system (CNS) [56]. Systematic reviews indicate that optimal ascorbate levels are associated with preserved memory function, whereas deficiencies are frequently observed in neurodegenerative conditions, including Alzheimer’s disease [56]. Preclinical studies further suggest that ascorbic acid supplementation may exert pro-cognitive effects, making it a promising adjunctive agent in the management of memory impairment and neurodegenerative disorders [57].

### 4.3. Vitamin A (Retinol)

Retinol, an exogenous fat-soluble vitamin, serves as an essential precursor to retinoic acid (RA), a key morphogenetic signaling molecule. The mechanism of RA action is based on the modulation of gene transcription through binding to specific nuclear receptors—retinoic acid receptors (RAR) and retinoid X receptors (RXR). This process is critical for neurogenesis, induction of neuronal differentiation, axial patterning of the central nervous system (CNS), and stimulation of motor axon outgrowth, playing a fundamental role both during embryonic development and in regenerative processes [58]. With advancing age, reduced bioavailability of vitamin A becomes an important risk factor for cognitive impairment. Proper retinoic acid signaling is essential for maintaining the structural and functional integrity of neurons in adulthood. Dysregulation of this signaling pathway triggers a cascade of neurodegenerative changes, including motor neuron degradation and the development of pathological features characteristic of Alzheimer’s disease. Furthermore, disturbances in retinoid homeostasis within the nigrostriatal pathway may negatively affect dopaminergic neuron survival, linking vitamin A metabolism to the etiopathogenesis of Parkinson’s disease [59]. Recent scientific findings further expand the role of vitamin A to its involvement in the microbiota–gut–brain axis. A synergistic relationship has been demonstrated between vitamin A intake and dietary fiber in the prevention of Alzheimer’s disease. Retinoic acid plays a crucial role in maintaining intestinal barrier integrity and modulating immune responses within the gastrointestinal tract. The interaction between retinoids and gut microbiota metabolites may effectively reduce low-grade inflammation and decrease amyloid plaque accumulation in the brain [60].

### 4.4. Vitamin E (Tocopherol)

Vitamin E, a key fat-soluble micronutrient, functions as the principal lipophilic antioxidant in the human body. Its primary mechanism of action involves interruption of free radical chain reactions and inhibition of lipid peroxidation within neuronal cell membranes, which are rich in polyunsaturated fatty acids. Functionally, tocopherol serves as the hydrophobic counterpart to the antioxidant activity of ascorbic acid, complementing its protective effects. Adequate intake of vitamin E is essential for maintaining metabolic homeostasis, whereas clinical deficiency leads to severe neurological disturbances, including spinocerebellar ataxia, peripheral neuropathy, and progressive axonal degeneration [61]. Recent scientific evidence has provided new insights into the influence of vitamin E on molecular aspects of nervous system aging. A key component of its action is the modulation of gene expression associated with aging processes. Vitamin E has been shown to downregulate the expression of cellular senescence markers such as p16 and p21, thereby contributing to the attenuation of neurodegenerative processes at the molecular level [62].

### 4.5. Vitamin K (Phylloquinone)

The term “vitamin K” refers to a group of structurally related compounds, among which phylloquinone (vitamin K_1_) and the family of menaquinones (collectively referred to as vitamin K_2_) are considered biologically most significant. Although these vitamins have traditionally been associated with hemostasis and bone metabolism, a growing body of evidence highlights their important role in maintaining central nervous system (CNS) homeostasis and in the prevention of neurodegenerative disorders [63]. Particular attention has been given to vitamin K_2_, which exhibits strong antioxidant and anti-inflammatory properties. This isoform plays a critical role in preserving cellular integrity by inhibiting inflammatory cascades and promoting the repair of mitochondrial damage induced by oxidative stress [64]. As a modulator of epigenetic processes, vitamin K_2_ may influence gene expression patterns associated with brain health. Through its capacity to protect mitochondrial function and regulate epigenetic mechanisms, vitamin K_2_ emerges as a promising factor in strategies aimed at preventing neuronal degeneration and preserving cognitive function [63].

### 4.6. Vitamin D (Cholecalciferol)

Within the central nervous system, vitamin D functions as a neurosteroid, exerting its effects on target cells through the activation of both classical genomic pathways—mediated by the vitamin D receptor (VDR)—and rapid non-genomic responses. This dual mechanism enables precise modulation of neuronal and glial cell physiology [65]. Clinically, vitamin D is recognized as a key neuroprotective factor, and its deficiency has been associated with the progression of neurodegenerative diseases, including Parkinson’s disease. Its pleiotropic actions include regulation of neurotrophic factor synthesis and protection of dopaminergic neurons, making it an essential component in maintaining functional brain integrity and preventing neural degeneration [65]. An important aspect of vitamin D biological activity is its role in maintaining neurological homeostasis by supporting repair and regenerative processes. Vitamin D plays a critical role in remyelination by stimulating oligodendrocyte differentiation and preserving myelin sheath integrity. Moreover, it exhibits strong antioxidant properties, protecting neural cells against oxidative stress-induced cytotoxicity and modulating immune responses within the central nervous system, thereby preventing chronic neuroinflammation [66].

### 4.7. Coenzyme Q10 (Ubiquinone)

Coenzyme Q10 (CoQ10) is an essential fat-soluble cofactor that plays a critical role in the mitochondrial electron transport chain. As a key component of cellular bioenergetics, CoQ10 mediates electron transfer from complexes I and II to complex III, a process required for efficient adenosine triphosphate (ATP) synthesis. Beyond its function in oxidative phosphorylation, CoQ10 exhibits potent antioxidant properties, protecting membrane lipids and mitochondrial DNA from damage induced by reactive oxygen species (ROS) [67]. Physiological levels of CoQ10 in brain tissue and other organs progressively decline with age, weakening antioxidant defenses and impairing neuronal bioenergetic capacity. Deficiency of this cofactor has been closely associated with the etiopathogenesis of several neurological disorders, including multiple sclerosis, Alzheimer’s disease, and Parkinson’s disease. In these conditions, mitochondrial dysfunction initiates a neurodegenerative cascade in which reduced CoQ10 availability exacerbates oxidative stress and promotes neuronal apoptosis [67]. Due to its ability to stabilize mitochondrial membranes, restore respiratory chain function, and attenuate neuroinflammation within the central nervous system, CoQ10 represents a promising target in the development of therapeutic strategies aimed at modifying the course of neurodegenerative diseases [67,68].

## 5. Synergistic Interactions Between Vitamins and Coenzyme Q10 in the Nervous System

Interactions between vitamins are often an overlooked element in the regulation of nervous system function. However, it is precisely the interactions among vitamins that determine the efficiency of many metabolic processes [45,69,70,71,72,73,74,75,76]. Due to its high oxygen consumption [77], the predominance of lipids susceptible to peroxidation, and the presence of redox-active metals combined with relatively low endogenous antioxidant activity [78], the brain is particularly vulnerable to oxidative stress [77,79]. Moreover, oxidative stress, inflammatory processes, and mitochondrial dysfunction mutually reinforce each other, thereby exacerbating the pathophysiological processes involved in neurodegenerative diseases [78,80,81,82,83,84]. Antioxidant vitamins such as vitamin E and vitamin C [69], as well as coenzyme Q10, exhibit synergistic activity [69,73,74]. Vitamin E neutralizes lipid peroxyl radicals and is oxidized to the tocopheroxyl radical [70], which is subsequently restored to its active form by both vitamin C [69,70] and coenzyme Q10 [74]. During this process, Q10 is oxidized to ubisemiquinone, which is then reduced back to its active form by vitamin C. This cycle of transformations inhibits the production of lipid peroxyl radicals, thereby protecting circulating lipoproteins, cellular membrane structures, and mitochondrial DNA [74]. UBIAD1 is an antioxidant enzyme that catalyzes the biosynthesis of coenzyme Q10 and vitamin K in the Golgi apparatus membrane and mitochondria, respectively, confirming the synergistic action of these compounds. Preclinical studies suggest that by increasing the synthesis of Q10 and vitamin K, UBIAD1 enhances their antioxidant potential. As a result, oxidative stress is reduced, ferroptosis is inhibited, and cellular as well as mitochondrial membranes are stabilized [84]. Additionally, it is suggested that coenzyme Q10 and vitamin D may reduce inflammation and oxidative stress while supporting proper mitochondrial function [75]. A clinical study has also demonstrated that combined supplementation with vitamins D and K improves markers of oxidative stress [70]. Vitamin B_9_ (folate) and vitamin B_12_ are key vitamins required for the proper functioning of one-carbon metabolism, which consists of two interrelated cycles: the folate cycle and the methionine cycle. These cycles converge at the level of methionine synthase (MTR), a vitamin B_12_-dependent enzyme. The 5,10-methylene-tetrahydrofolate (5,10-methylene-THF) produced in the folate cycle is essential for DNA and RNA synthesis, while methyl groups are transferred to the methionine cycle, where they participate in the remethylation of homocysteine. The MTR–B_12_ complex converts homocysteine into methionine, which is subsequently transformed into S-adenosylmethionine (SAM) [73]. SAM serves as a key methyl group donor in numerous methylation reactions, particularly in the central nervous system (CNS), where it participates in the synthesis of phospholipids and biogenic amines [45]. After donating a methyl group, SAM is converted into S-adenosylhomocysteine (SAH), which is subsequently hydrolyzed back to homocysteine by the enzyme adenosylhomocysteinase [72]. Disruptions in DNA methylation may lead to neurological disorders, developmental delay, and impaired cognitive function. Deficiency of either folate or vitamin B_12_ results in elevated homocysteine levels [45], which exhibit neurotoxic properties. Moreover, clinical studies suggest that excessive folic acid supplementation may mask vitamin B_12_ deficiency and contribute to increased homocysteine concentrations [76]. Elevated homocysteine levels positively correlate with β-amyloid (Aβ) accumulation, impaired neurotransmitter synthesis, inflammation, and increased production of reactive oxygen species (ROS) in mitochondria. These effects ultimately contribute to cognitive decline and the development of dementia [85]. Understanding the interactions between vitamins and coenzyme Q10 is essential for the development of effective preventive strategies and supportive therapies in neurodegenerative diseases. Nevertheless, many of the relationships between vitamins and Q10 remain insufficiently understood, highlighting the need for further research and more detailed investigations.

## 6. Role of Selected Vitamins in the Development of Neurodegenerative Diseases

### 6.1. Vitamins A, C, and E

Vitamins A, C, and E primarily exert anti-inflammatory and antioxidant effects. They also prevent the abnormal aggregation of β-amyloid (Aβ), neurofibrillary tangles (NFTs), and α-synuclein (α-syn) [86,87,88,89,90,91,92,93,94,95,96,97,98,99], thereby potentially reducing the clinical manifestations of neurodegenerative diseases. In patients with Alzheimer’s disease (AD), decreased serum concentrations of vitamins A, C, and E have been reported and are associated with dementia and reduced cognitive performance [86]. Li et al. evaluated the effects of supplementation with vitamins E, C, and β-carotene on cognitive function in 300 elderly individuals. After a 16-week intervention, cognitive performance was assessed using the Hasegawa Dementia Scale (HDS) and the Mini-Mental State Examination (MMSE), while radioimmunoassay (RIA) was used to measure Aβ and estradiol (E2) levels. The study demonstrated that supplementation improved cognitive function, reduced Aβ levels, and increased E2 concentrations [88]. In an in vivo experimental study, administration of the active vitamin A metabolite all-trans retinoic acid (ATRA) to APP/PS1 transgenic mice with AD illustrated its neuroprotective properties. ATRA reduced Aβ production and deposition, tau phosphorylation, microglial and astrocyte activation, neuronal degeneration, and improved cognitive function in mice [89]. Antioxidant vitamins also exhibit neuroprotective effects in Parkinson’s disease (PD) [90]. Reduced vitamin C levels have been observed in PD patients. Vitamin C suppresses glutamate-induced excitotoxicity, modulates GABA receptor activity, and inhibits α-syn oligomerization by preventing DOPAL oxidation [90,91]. Additionally, vitamin C enhances L-dopa absorption, increasing therapeutic efficacy [91]. Higher dietary vitamin E intake has been associated with lower PD risk, and experimental models indicate that vitamin E improves synaptic plasticity and mitochondrial function [92]. Vitamin A supplementation has also been shown to increase dopaminergic neuron survival and improve motor function in animal models [93]. However, some studies report no clear association between antioxidant vitamin levels and PD risk or progression [94,95]. Divergent findings regarding the effects of antioxidant vitamins on the course of Parkinson’s disease (PD) may result from the considerable heterogeneity of the methods used. A summary of the studies investigating the effects of antioxidant vitamins (A, C, and E) in neurodegenerative diseases is presented in Table 1. The analyzed studies include in vitro and in vivo models, as well as case–control and observational studies involving both humans and animals, which inherently generates substantial variability in the obtained results. Additionally, individual experiments differed in the duration of interventions, vitamin dosages, and the forms of administered vitamins, all of which may influence the magnitude and direction of the biological response. Individual factors also play an important role, including nutritional status, vitamin metabolism, genetic background, and disease stage, which may further confound the overall outcomes of analyses. Consequently, some studies indicate a potential neuroprotective effect of vitamins, whereas others show no association with the risk or progression of PD. To obtain more conclusive results, further studies with standardized methodologies are required [92,93,94,95]. In amyotrophic lateral sclerosis (ALS), vitamin C may prevent motor neuron loss and reduce excessive AMPA receptor activation [91], while vitamin E modulates p38 MAPK and JNK signaling pathways and improves motor function [87]. Some evidence suggests that these vitamins may be associated with reduced mortality in ALS [91,96,97], although conflicting findings remain [98]. Continued research is therefore necessary to clarify their role in the progression of neurodegenerative diseases [87,99]. It is also worth noting that the therapeutic effects observed in clinical studies are typically associated with high vitamin doses, often several times higher than the recommended dietary intake but still within the Tolerable Upper Intake Level (UL) established by the European Food Safety Authority (EFSA) [88,100]. This raises the question of whether preventive strategies against neurodegenerative diseases can be effectively achieved through diet alone, or whether vitamin supplementation at pharmacological doses is required. Further research is needed to determine the optimal intake levels of individual vitamins for the prevention of neurodegeneration.

### 6.2. Vitamins B_1_, B_12_, and Folic Acid

B vitamins play a crucial role in maintaining proper nervous system function, and their deficiency may contribute to neurodegenerative processes [86]. In Alzheimer’s disease (AD), reduced levels of B-complex vitamins have been observed [86]. These vitamins are associated with reduced β-amyloid (Aβ) accumulation [99] and decreased tau hyperphosphorylation [86,101]. A cross-sectional observational study involving 2422 older adults demonstrated that higher dietary intake of vitamin B_1_ was correlated with better cognitive performance [102]. A randomized, single-blind, placebo-controlled trial investigating 6-month supplementation with vitamin B_12_ (50 μg/day) and folic acid (1.2 mg/day) in AD patients showed improvements in cognitive function, reduced inflammation, decreased homocysteine and S-adenosylhomocysteine levels, and increased S-adenosylmethionine concentrations [103]. However, some studies indicate that although folic acid and vitamin B_12_ supplementation may reduce homocysteine levels, they do not necessarily improve daily functioning in AD patients [104]. In PD, B vitamins such as B_12_ and B_1_ attenuate neuronal degeneration, oxidative stress, inflammation, and apoptosis, with vitamin B_12_ additionally inhibiting α-synuclein aggregation [105]. Elevated vitamin B_12_ levels at the time of PD diagnosis have been associated with a lower risk of developing dementia in later disease stages [106]. A cross-sectional study involving 148 patients with idiopathic PD confirmed that adequate folate and vitamin B_12_ levels were associated with improved motor function [107]. Additionally, Håglin et al. reported that insufficient dietary intake of vitamin B_1_ and folate 2–8 years prior to PD diagnosis was associated with olfactory dysfunction at the time of diagnosis [108]. B vitamins also influence ALS progression. Vitamin B1 deficiency has been linked to increased oxidative stress and intracellular calcium dysregulation, potentially contributing to disease initiation [97]. In a multicenter, double-blind, placebo-controlled phase III clinical trial, patients with ALS received intramuscular methylcobalamin (50 mg) once weekly for 16 weeks. Active vitamin B_12_ significantly slowed motor function decline in patients at early disease stages with mild progression [109]. In an SOD1 (G93A) mouse model, folic acid and vitamin B_12_ reduced plasma homocysteine levels, suppressed glial activation, decreased inflammation, and inhibited motor neuron loss [110]. Nevertheless, large-scale clinical trials have not confirmed a significant effect of folic acid supplementation on ALS progression [101]. In clinical studies in which a slowing of neurodegenerative disease progression was observed, B vitamins were administered at doses significantly exceeding the recommended daily intake established by the European Food Safety Authority (EFSA). Moreover, some studies used folate doses exceeding the tolerable upper intake level (UL), while no upper safe intake level has yet been established for vitamin B_12_ [100,103,109]. It should also be emphasized that therapeutically effective doses cannot be obtained through diet alone. To better systematize current knowledge regarding the differences between preventive and therapeutic vitamin doses, further studies are required to establish precise recommendations for vitamin supplementation in the context of neurodegenerative diseases. A summary of the studies investigating the effects of B vitamins in neurodegenerative diseases is presented in Table 2.

### 6.3. Vitamins D and K, and Coenzyme Q10

Vitamins D and K, as well CoQ10, are important neuroprotective factors that reduce both oxidative stress and inflammation observed in neurodegenerative disorders [99,111,112,113]. Vitamins D and K together with CoQ10 limit Aβ toxicity [99,113,114], which translates into improved synaptic plasticity [115] and cognitive performance [99,114]. Additionally, vitamins D and K inhibit α-synuclein (α-syn) aggregation [99,111], while vitamin K further reduces phosphorylated tau deposition [99]. Within microglia, provitamin D undergoes activation to its biologically active form, which modulates the synthesis of glial cell line-derived neurotrophic factor (GDNF) and nerve growth factor (NGF), both crucial for learning and memory processes [87]. A randomized, double-blind, placebo-controlled trial demonstrated that one-year supplementation with vitamin D at a dose of 800 IU/day in 210 patients with AD improved cognitive function and reduced levels of Aβ-related biomarkers [114]. Abuelezz et al. evaluated the effects of CoQ10 in a scopolamine-induced rat model of AD. Their findings showed that CoQ10 regulated key signaling pathways involved in neurodegeneration, reduced oxidative stress, and improved memory and cognitive performance [115]. A review of preclinical data suggests that vitamin K_2_ may exhibit antioxidant, anti-inflammatory, and mitochondria-supporting effects in models of Alzheimer’s disease (AD). However, clinical studies confirming these observations are still lacking [116]. Moreover, vitamin D exhibits clear neuroprotective properties toward dopaminergic neurons through microglial modulation [89] and inhibition of excitotoxic and apoptotic processes [111]. An analysis of 2866 individuals with PD indicated an association between adequate vitamin D levels and a lower risk of symptom progression and disease advancement [87]. Higher doses of supplemented vitamin D were associated with improved balance control and fewer falls in younger individuals with PD; however, similar benefits were not observed in older patients [111]. Protective effects on dopaminergic neurons have also been described for vitamin K [89], which has been reported at lower serum levels in PD patients in a case–control study [117]. Reduced CoQ10 levels have likewise been observed in PD and are associated with impaired motor function [112]. In a mouse model of PD, CoQ10 supplementation was correlated with reduced inflammation, increased expression of neurogenesis and angiogenesis markers, and greater dopaminergic neuron density [118]. Nevertheless, large randomized, double-blind, placebo-controlled trials have failed to demonstrate significant clinical benefits of CoQ10 supplementation in PD, despite promising earlier findings in smaller cohorts [119]. Vitamin D deficiency has been reported in ALS patients [89] and may contribute to greater disease severity [120]. The active form of vitamin D plays a crucial role in calcium homeostasis [111]. It has also been suggested that vitamin D improves motor performance in the SOD1-G93A mouse model of ALS. Furthermore, limited evidence indicates that adequate vitamin D levels may correlate with prolonged survival in ALS patients [120]. Conversely, analyses of ALS patients with severe vitamin D_3_ deficiency have shown that supplementation with various doses for six months did not improve motor dysfunction [121]. Based on current evidence, the mechanisms by which CoQ10 affects motor neurons and ALS progression remain unclear, highlighting the need for further research in this area [112]. Moreover, the current literature lacks clinical studies investigating the effects of vitamin K in amyotrophic lateral sclerosis (ALS), highlighting the need for further in-depth research in this area. Therapeutic effects of vitamins in neurodegenerative diseases have been confirmed in clinical studies only for vitamin D, whose dosing fell within both the recommended intake range and the tolerable upper intake level (UL) established by the European Food Safety Authority (EFSA) [100,114]. Additionally, the limited number of clinical studies prevents a comprehensive synthesis of the available data and the clear determination of potential therapeutic doses. This topic therefore requires further clinical investigations to establish precise and safe vitamin supplementation strategies. Table 3 summarizes studies on the effects of vitamins D, K, and Coenzyme Q10 in neurodegenerative diseases.

The relationship between therapeutic doses and recommended intake levels is presented in Table 4.

## 7. Supplementation in Prevention and Treatment

In the absence of fully effective causal therapies, research attention has increasingly shifted toward preventive and supportive strategies, among which the optimization of micronutrient status occupies a central position as a potential modulator of neurodegenerative diseases. Vitamins and metabolic cofactors, as essential regulators of enzymatic, metabolic, and signaling pathways, form a fundamental basis of central nervous system (CNS) homeostasis. Although vitamin supplementation is often regarded as a promising non-pharmacological strategy in combating neurodegeneration, it represents a highly complex field in which therapeutic optimism must be carefully weighed against clinical evidence and potential risks associated with excessive intake.

A key area of investigation concerns the impact of B-complex vitamins on cognitive function. Vitamin B_12_, vitamin B_6_, and folic acid have been shown to play a decisive role in lowering levels of homocysteine—an amino acid with well-documented neurotoxic properties. B vitamins may contribute to slowing the progression of mild cognitive impairment toward overt dementia [122]. In the systematic review conducted by [123], the authors confirmed that supplementation with B vitamins effectively reduces plasma homocysteine levels. In the studies included in the meta-analysis, this reduction was clear and consistent. However, the authors emphasized that the meta-analysis showed that lowering homocysteine levels through B vitamin supplementation did not correlate with a statistically significant improvement in cognitive test outcomes. Furthermore, studies lasting from 6 to 24 months indicated that despite the reduction in homocysteine concentration, no significant inhibition of dementia symptoms at the population level was observed. The results of the meta-analysis suggest that vitamin supplementation may be most effective in individuals with elevated baseline homocysteine levels or in patients with confirmed vitamin B12 and folate deficiencies [123]. In the context of Alzheimer’s disease, studies on vitamin E have demonstrated its ability to slow functional decline. Administration of 2000 IU/day of alpha-tocopherol significantly delayed deterioration in activities of daily living compared with placebo, supporting its role as an adjunctive therapeutic option [124]. Vitamin D, acting as a neurosteroid, influences a broad range of neuropsychiatric processes, ranging from the modulation of neurotransmission to the reduction in neuroinflammation, thereby positioning it as an important factor in maintaining central nervous system (CNS) homeostasis [125]. A meta-analysis including 24 clinical trials [126] demonstrated that vitamin D supplementation significantly affects overall cognitive function. The review clearly indicates that the effectiveness of supplementation depends on baseline vitamin D levels prior to treatment. Patients with an initial vitamin D deficiency below 20 ng/mL (50 nmol/L) derived the greatest benefit from supplementation. Correction of vitamin D deficiency was associated with improved carbohydrate metabolism and lipid profile, modulation of inflammatory responses, support of the immune system, and beneficial effects on bone health. In contrast, individuals with normal baseline vitamin D levels did not experience additional benefits from supplementation. The meta-analysis did not demonstrate a significant effect on specific brain functions, including short-term memory, executive functions, or information processing speed. Instead, improvements were observed primarily in overall intellectual performance. The publication also provides evidence for the neuroprotective mechanisms of vitamin D, including the reduction in amyloid plaque formation, anti-inflammatory effects, and the regulation of calcium homeostasis in neuronal cells [126].

Despite these potential benefits, growing evidence highlights the risks associated with uncontrolled supplementation. A notable example is the “vitamin B6 paradox,” whereby excessive pyridoxine intake may induce functional deficiency and neurotoxicity, manifesting as sensory neuropathy due to inhibition of pyridoxal phosphate-dependent enzymes [127]. Another concern involves the imbalance between folic acid and vitamin B_12_. In older adults, elevated folate levels combined with cobalamin deficiency may mask hematological symptoms such as anemia while simultaneously accelerating cognitive decline and exacerbating neurological damage [128]. Significant methodological limitations also persist. A major challenge lies in the discrepancy between observational studies and randomized clinical trials. Long-term investigations, including decade-long follow-up studies in male populations, have often failed to demonstrate significant differences in cognitive outcomes between multivitamin and placebo groups [129]. These findings suggest that supplementation initiated in advanced age may represent a delayed intervention, given that neurodegenerative processes begin many years before the onset of clinical symptoms.

## 8. Current Challenges and Future Perspectives

Preclinical data confirm the anti-inflammatory, antioxidant, and mitochondria-supporting effects of vitamin K [116,130,131], providing a theoretical basis for the assumption that this compound may offer health benefits in individuals with neurodegenerative diseases. However, the current state of knowledge allows conclusions to be drawn only from preclinical models, the results of which do not always translate directly to human populations. Moreover, no studies to date have evaluated the effects of vitamin K in preclinical models of amyotrophic lateral sclerosis (ALS). The lack of clinical studies assessing therapeutic doses of vitamin K and their impact on neurodegenerative diseases such as Alzheimer’s disease (AD), Parkinson’s disease (PD), and ALS highlights the need for further systematic research in this field, as well as the initiation of well-designed clinical trials.

Neurodegenerative pathology in Parkinson’s disease progresses over time and gradually affects increasingly larger areas of the brain. However, the only robust evidence regarding the presence and toxic effects of DOPAL currently relates to the substantia nigra pars compacta (SNpc) [32] and part of the putamen. There are no data confirming the presence of DOPAL or describing its mechanisms of action in other regions of the striatum, such as the caudate nucleus or the prefrontal cortex [33]. It has been suggested that degeneration of dopaminergic neurons in the ventral tegmental area (VTA) may contribute to the development of non-motor symptoms of PD. However, despite the relatively large number of dopaminergic neurons present in the VTA [132], no correlation has yet been demonstrated between this brain region and the presence or activity of DOPAL. Therefore, further research into the mechanisms of DOPAL neurotoxicity is required, as a better understanding of these mechanisms may provide a basis for the development of novel therapeutic strategies for Parkinson’s disease.

Despite considerable genomic and physiological similarities between rodents and humans, important differences exist in metabolic processes unrelated to pharmacokinetics. These differences may determine whether a given compound induces toxicity or alters biomarker levels. For example, rodents synthesize vitamin C, degrade uric acid into allantoin, and produce specific bile acids, distinguishing them from humans [133]. In addition, humans exhibit a higher Firmicutes-to-Bacteroidetes ratio than rodents. The human microbiota is dominated by Bacteroides, whereas mice are dominated by representatives of the S24-7 family, and rats by Prevotella. Furthermore, lactate levels in feces are higher in rodents than in humans, while acetate levels show the opposite pattern [134]. These interspecies differences may limit the direct translation of some experimental findings. Therefore, interpretation of preclinical data requires caution and consideration of species-specific physiological differences. Understanding interspecies differences in vitamin metabolism is essential for improving the translation of findings from preclinical studies to clinical research, highlighting the need for further investigation in this area.

Multimodal interventions, such as combining vitamin supplementation with dietary fiber, plant bioactive compounds, prebiotics, probiotics, or microbiome-targeted therapies, may be particularly relevant in the context of neurodegenerative diseases. The gut microbiota communicates with the brain via the vagus nerve, enabling bidirectional signaling between these systems. Consequently, changes in one environment may influence the other. It is suggested that key microbial metabolites, such as short-chain fatty acids (SCFAs), may play an important role in brain regulation. SCFAs may affect the brain through direct humoral signaling, indirect hormonal mechanisms, modulation of immune responses and neuronal pathways, as well as through interactions with G protein-coupled receptors and histone deacetylases. Alterations in the gut microbiota profile have been observed in patients with neurodegenerative diseases and are often associated with enhanced inflammatory processes. Therefore, therapies aimed at modulating the microbiome appear promising, although their effectiveness has not yet been definitively confirmed and requires further clinical investigation [135]. Dietary micronutrients, including vitamins, may influence the composition and functionality of the microbiome. Conversely, the microbiome affects their bioavailability, for example through vitamin synthesis and regulation of nutrient absorption. Thus, microbiome-based interventions may represent an effective strategy for addressing vitamin deficiencies [136]. Dietary fiber also plays an important role, as it positively modulates the gut microbiota profile and increases SCFA production [137]. Moreover, enriching therapeutic approaches with bioactive plant compounds may enhance antioxidant capacity and provide additional neuroprotective effects [138]. A study by Benincasa et al. demonstrated that the combination of curcumin, vitamin E, and vitamin C exerted stronger antioxidant effects on erythrocytes than each compound administered individually [139]. This experiment highlights the synergistic interactions between vitamins and plant bioactive compounds, providing a solid foundation for further studies that may contribute to the development of novel therapeutic strategies against neurodegenerative diseases. The search for additional synergistic interactions between various compounds and biological systems encourages the development of innovative multimodal interventions, which may prove more effective than single-agent therapies.

## 9. Conclusions

Neurodegenerative diseases such as Alzheimer’s disease, Parkinson’s disease, and amyotrophic lateral sclerosis are characterized by progressive neuronal loss, mitochondrial dysfunction, and chronic neuroinflammation. Clinical and animal studies suggest that supplementation with selected vitamins—particularly B-complex vitamins (B_1_, folic acid, B_12_), as well as vitamins A, C, D, E, and K and coenzyme Q10—may alleviate symptoms and potentially slow the progression of neurodegeneration. These micronutrients contribute to mitochondrial energy production, protection against oxidative stress, neurotransmitter synthesis, and the modulation of inflammatory responses within the nervous system, supporting their potential therapeutic relevance.

However, further research is required to establish precise dosing strategies and determine the most effective vitamin forms tailored to specific disease states. Despite well-documented protective mechanisms, the clinical effectiveness of vitamin supplementation in preventing or treating neurodegenerative disorders faces significant methodological challenges and potential clinical risks. Evidence indicates that the efficacy of vitamin-based interventions strongly depends on the patient’s baseline metabolic status and the stage of disease progression. Interventions initiated in advanced stages of neurodegeneration often prove ineffective due to irreversible structural brain damage.

Future therapeutic strategies should move beyond generalized supplementation toward precision metabolic medicine, emphasizing standardized dosing, the use of highly bioavailable forms, and careful biochemical monitoring. Only an integrated approach—combining early diagnosis with targeted administration of specific metabolic cofactors—will allow for the full realization of the neuroprotective potential of vitamins in modifying the course of neurodegenerative diseases and preserving cognitive function in aging individuals.

## Figures and Tables

**Table 1:** Studies on the effects of antioxidant vitamins (A, C, and E) in neurodegenerative diseases.

Ref.	Intervention (Dose and Duration)	Study Model	Main Outcomes
[88]	Vitamin E (200 mg/day); Vitamin C (300 mg/day); β-carotene (16.7, 8.4, 5.6, or 0 mg/day); 16 weeks	Prospective study; *n* = 300 (elderly without severe comorbidities)	↑ Cognitive function (HDS, MMSE); ↓ Aβ; ↑ Estradiol (E2)
[89]	All-trans retinoic acid (ATRA), 20 mg/kg, 3×/week; 8 weeks	In vivo; APP/PS1 double-transgenic mice *n* = 24 (12 AD, 12 controls)	↓ APP phosphorylation; ↓ Tau phosphorylation; ↓ Microglial and astrocyte activation; ↓ Neuronal degeneration; ↑ Cognitive function
[92]	α-Tocopherol (100 μM, ~100 min); Trolox (100 μM, ~100 min);	Case–control study (dietary questionnaire); *n* = 200 (100 PD, 100 controls);	Higher dietary vitamin E intake was associated with a lower risk of PD
	α-Tocopherol (100 mg/kg, 7 days); Trolox (5 mg/kg, 7 days)	ex/in vitro; PINK1−/−model	Vitamin E reversed synaptic plasticity impairments
[93]	Vitamin A (20 IU/g vs. control 5 IU/g); 8 weeks	In vivo; rat model of PD; N = 66	↑ Dopaminergic neuron number; ↑ Striatal D2 and RXR receptor expression; ↑ Motor performance
[94]	-	Observational study; *n* = 43 (27 PD, 16 controls)	No differences in serum vitamin A and E; ↑ Vitamin C in PD; No correlation with PD progression
[95]	-	Cohort study; *n* = 1036 PD patients	No association between antioxidant vitamin intake and PD risk
[98]	-	Observational study; *n* = 624 (202 ALS, 214 ALS-mimic disorders, 208 controls)	↑ Vitamin A and E; ↓ Vitamin C in ALS; No correlation with ALS progression

Abbreviations: Aβ—beta-amyloid; ALS—amyotrophic lateral sclerosis; APP—amyloid precursor protein; ATRA—all-trans retinoic acid; E2—estradiol; HDS—Hasegawa Dementia Scale; MMSE—Mini-Mental State Examination; PD—Parkinson’s disease; RXR—retinoid X receptor; ↑ increase; ↓ decrease.

**Table 2:** Comparison of studies on B vitamins in neurodegenerative diseases.

Ref.	Intervention (Dose and Duration)	Study Model	Main Outcomes
[102]	—	Bidirectional Mendelian randomization analysis	No effect of folic acid intake on ALS progression
[102]	—	Cross-sectional observational study; *n* = 2422 adults ≥ 60 years	Higher dietary vitamin B_1_ intake associated with ↑ cognitive function
[103]	Folic acid 1.2 mg/day + Vitamin B_12_ 50 µg/day; 6 months	Randomized, single-blind, placebo-controlled trial; *n* = 101 AD patients	↑ Cognitive function; ↓ Inflammation; ↓ Homocysteine; ↓ S-adenosylhomocysteine; ↑ S-adenosylmethionine
[104]	Folic acid + Vitamin B_12_ (varied doses across RCTs); 6 months	Meta-analysis of randomized controlled trials; *n* = 780 AD patients	↓ Homocysteine; ↑ Cognitive function; No significant improvement in daily functioning
[106]	—	Cohort study; *n* = 301 PD patients	Higher vitamin B_12_ serum levels associated with ↓ risk of dementia in later PD stages
[107]	—	Cross-sectional study; *n* = 148 idiopathic PD patients	Vitamin B_12_ serum levels and immune markers correlated with motor function
[108]	—	Prospective case–control study; *n* = 618 PD patients	Low dietary B_1_ and folate intake associated with ↑ olfactory dysfunction at diagnosis
[109]	Methylcobalamin 50 mg IM twice weekly; 16 weeks	Multicenter, double-blind, placebo-controlled phase III RCT; *n* = 130 ALS patients	↓ Rate of motor function decline; Treatment safe during intervention
[110]	Folic acid 4 mg/kg/day ± Vitamin B_12_ 0.2 mg/kg/day (from week 6 of life)	In vivo study; SOD1G93A transgenic mice; N = 48	↓ Homocysteine; ↓ Inflammation; ↓ Glial activation; ↓ Motor neuron loss

Abbreviations: AD—Alzheimer’s disease; ALS—amyotrophic lateral sclerosis; PD—Parkinson’s disease; RCT—randomized controlled trial; IM—intramuscular; SOD1^G93A—superoxide dis-mutase 1 (G93A) transgenic mouse model; NaCl—sodium chloride; SAM—S-adenosylmethionine; SAH—S-adenosylhomocysteine; ↑ increase; ↓ decrease.

**Table 3:** Comparison of studies on vitamins D and K and Coenzyme Q10 in neurodegenerative diseases.

Ref.	Intervention (Dose and Duration)	Study Model	Main Outcomes
[114]	Vitamin D 800 IU/day; 1 year	Randomized, double-blind, placebo-controlled trial; *n* = 210 AD patients	↑ Cognitive function; ↓ Aβ levels
[115]	CoQ10 50 or 100 mg/kg/day; 6 weeks	In vivo study; scopolamine-induced cognitive impairment rat model; *n* = 60	Regulation of key neurodegeneration pathways; ↓ Oxidative stress; ↑ Memory and cognitive function
[116]	—	Case–control study; *n* = 188 (93 PD patients, 95 controls)	↓ Vitamin K serum levels in PD; Low vitamin K associated with immune dysregulation and PD progression
[117]	CoQ10 1.5 or 2.4 µg/day; ~4 weeks	In vivo study; rat model of PD; *n* = 17 ((3 no-treatment, 3 oral-Q10, 3 Alzet-PBS, 3 Alzet-low-Q10, 5 Alzet-high-Q10)	↑ Dopaminergic neuron survival; ↑ Neurogenesis and angiogenesis markers; ↓ Striatal inflammation
[120]	Cholecalciferol 50,000; 75,000; or 100,000 IU/month; 6 months	Randomized cohort study; *n* = 48 ALS patients (34 deficient < 20 ng/mL; 14 insufficient)	No improvement in motor dysfunction regardless of dose

Abbreviations: AD—Alzheimer’s disease; ALS—amyotrophic lateral sclerosis; PD—Parkinson’s disease; CoQ10—coenzyme Q10; IU, international units; Aβ—beta-amyloid; 25(OH)D—25-hydroxyvitamin D; ↑ increase; ↓ decrease.

**Table 4:** Comparison of therapeutic doses used in studies with recommended intakes and upper intake levels (UL) for adults according to the EFSA.

Ref.	Vitamin	Therapeutic Dose	Recommended Intake	UL (EFSA)	Summary
[88]	Vitamin E	200–300 mg/day	11 mg/day (F); 13 mg/day (M)	300 mg/day	The therapeutic dose of vitamin E was several times higher than the recommended intake but remained below the EFSA UL, indicating a safe range of intake.
[88]	Vitamin C	200–300 mg/day	95 mg/day (F); 110 mg/day (M)	Not established	The therapeutic dose was approximately three times higher than the recommended intake; however, since the EFSA has not established a UL for vitamin C, it is considered safe.
[102]	Vitamin B9 (Folate)	1.2 mg/day	330 µg/day	1000 µg/day	The therapeutic dose exceeded the recommended intake and slightly surpassed the UL established by the EFSA.
[102]	Vitamin B12	50 µg/day	4 µg/day	Not established	The therapeutic dose was over 12 times higher than the recommended intake; however, no UL has been established for vitamin B12 by the EFSA.
[109]	Vitamin B12 (Methylcobalamin)	50 mg/day	4 µg/day	Not established	The methylcobalamin dose was significantly higher than the recommended intake but remains within the safety range due to the absence of an EFSA UL.
[114]	Vitamin D	800 IU/day	15 µg/day	100 µg/day	The therapeutic dose was close to the recommended intake and remained below the EFSA UL, indicating a safe supplementation level.

UL—Tolerable Upper Intake Level; EFSA—European Food Safety Authority; F—female; M—male.

## Data Availability

Not applicable.

## Abbreviations

The following abbreviations are used in this manuscript:

AD	Alzheimer’s disease
ALS	Amyotrophic lateral sclerosis
AMPA	α-amino-3-hydroxy-5-methyl-4-isoxazolepropionic acid
APP	Amyloid precursor protein
ATRA	All-trans retinoic acid
Aβ	Beta-amyloid
BACE-1	Beta-site APP cleaving enzyme 1
CNS	Central nervous system
CoQ10	Coenzyme Q10
DOPAL	3,4-Dihydroxyphenylacetaldehyde
DPR	Dipeptide repeat proteins
EAAT2	Excitatory amino acid transporter 2
E2	Estradiol
GABA	Gamma-aminobutyric acid
GDNF	Glial cell line-derived neurotrophic factor
HDS	Hasegawa Dementia Scale
IM	Intramuscular
IU	International units
LB	Lewy bodies
LRP1	Low-density lipoprotein receptor-related protein 1
MMSE	Mini-Mental State Examination
NaCl	Sodium chloride
NGF	Nerve growth factor
NFT	Neurofibrillary tangles
PD	Parkinson’s disease
RA	Retinoic acid
RAGE	Receptor for advanced glycation end products
RAR	Retinoic acid receptor
RCT	Randomized controlled trial
ROS	Reactive oxygen species
RXR	Retinoid X receptor
SAH	S-adenosylhomocysteine
SAM	S-adenosylmethionine
SNpc	Substantia nigra pars compacta
SOD1^G93A	Superoxide dismutase 1 (G93A) transgenic mouse model
SVCT2	Sodium-dependent vitamin C transporter 2
TPP	Thiamine pyrophosphate
VDR	Vitamin D receptor
α-syn	Alpha-synuclein
